# Supplementary material for: Biocides with Controlled Degradation for Environmentally Friendly and Cost-Effective Fecal Sludge Management
Source: Biology (Basel). 2022 Dec 26;12(1):45. doi: 10.3390/biology12010045 (PMC9855048; doi:10.3390/biology12010045)
Supplement: Supplementary file 1 [file biology-12-00045-s001.zip › biology-2069571-supplementary.docx]

**Table S1: The cost of using biocidal preparations for the treatment of fecal sludge.**

| **Biocide** | **Dosage per 1000 liters of FS, mL (g)** | **Cost of 1 kg (L) of biocide, $** | **Treatment cost of 1000 liters of FS, $** |
| --- | --- | --- | --- |
| ‘’Latrina’’ | 700 | 3.0* | 2.1 |
| DBNPA | 200 | 1.5-2.5 [1] | 0.3-0.5 |
| Bronopol (B) | 30-60 | 1.90 [2] | 0.06-0.11 |
| Sharomix (сoncentrate) (SH) | 55 | 1.5-3.0 [3] | 0.08-0.17 |
| Sodium Percarbonate (P) | 6,000 | 0.2-0.4 [4] | 1.2-2.4 |
| Silver citrate (SC) | 5,000-20,000 | 5.0 [5] | 25-100 |
| Dehydroacetic acid, sodium salt (DAN) | 20,000-55,000 | 1.8-3.6 [6] | 36-198 |

Note: * - information is provided by the manufacturer (Limited Liability Company «Rail Chemical», Russia)

References

1. <https://www.alibaba.com/product-detail/Dbnpa-Dbnpa-Biocide-Water-Treatment-Chemical_60702852680.html?spm=a2700.7724857.normal_offer.d_image.16dd2ad7suAx1o&s=p>
2. <https://www.alibaba.com/product-detail/BRONOPOL-52-51-7-2-bromo_60161726874.html?spm=a2700.7724857.topad_classic.d_image.79596392S1Fqlk&fullFirstScreen=true>
3. <https://www.alibaba.com/product-detail/methylchloroisothiazolinone-methylisothiazolinone-mci-mi-60697190980.html?spm=a2700.8699010.29.1.6e006fb7rgrKjU>
4. <https://www.alibaba.com/p-detail/Detergent-1756940392.html?spm=a2700.galleryofferlist.normal_offer.d_title.4fe96d18Kd2VwP>
5. <https://www.alibaba.com/product-detail/Factory-Price-Sell-Silver-Citrate-Powder_1600101095130.html>
6. https://www.alibaba.com/product-detail/Sodium-Dehydroacetate-Sodium-Dehydroacetate-Sodium-Dehydroacetate_1600245139757.html?spm=a2700.galleryofferlist.normal_offer.d_title.30fd5387AeqxEa
